# Evaluation of whole-body MRI for cancer early detection in Li-Fraumeni syndrome

**DOI:** 10.1136/jmg-2025-110704

**Published:** 2025-06-30

**Authors:** Peter Sodde, Zerin Hyder, Sarah Pugh, Fiona Lalloo, Richard Martin, Calvin Soh, Jawad Naqvi, Richard Whitehouse, D Gareth Evans, Emma Roisin Woodward

**Affiliations:** 1 Division of Cancer Sciences, Faculty of Medicine, Biology and Health Sciences, The University of Manchester, Manchester, UK; 2 Manchester Centre for Genomic Medicine (MCGM), Manchester University Foundation Trust, Manchester, UK; 3 Centre for Life, Newcastle upon Tyne, UK; 4 Department of Radiology, Manchester University Foundation Trust, Manchester, UK; 5 Division of Evolution, Infection and Genomics, Faculty of Medicine, Biology and Health Sciences, University of Manchester, Manchester, UK

**Keywords:** Early Diagnosis, Heredity

## Abstract

Li-Fraumeni syndrome (LFS) is a high-risk hereditary cancer predisposition syndrome affecting 1 in 5000 individuals. Current standard of care in adults includes annual whole-body MRI (WB-MRI) and MRI brain (MRB) surveillance to enable early cancer detection. We performed a retrospective single-centre study of adults with *TP53* pathogenic germline variants or proven somatic mosaicism undergoing annual WB-MRI surveillance between January 2012 and January 2024, and MRB surveillance between August 2017 and January 2024. 325 WB-MRI scans were performed in 75 individuals. 17 cancers were diagnosed in 16 individuals. Nine out of 17 cancers were WB-MRI detected (7/9 had stage 1/2 disease). Benign incidental findings were identified in 89/325 (27.4%) of WB-MRI scans, prompting 53 additional investigations. As a stand-alone surveillance tool, WB-MRI demonstrated a pan-cohort specificity of 95.5%, negative predictive value of 97.4% and sensitivity of 42.9%. 32 individuals underwent 53 MRB scans detecting one cancer. We report the findings from the longest and largest single-centre experience of WB-MRI surveillance for cancer early detection in adults with LFS, demonstrating a high and acceptable level of cancer exclusion but modest sensitivity with WB-MRI prompting a significant number of additional investigations.

## Introduction

 Li-Fraumeni syndrome (LFS) is a high-risk hereditary cancer predisposition syndrome arising from pathogenic germline variants (PGVs) of the *TP53* gene, associated with a cancer risk of 80% by age 70 years^1 2^ and prevalence of 1 in 5000 individuals,^3^ with metachronous primary tumours seen in over 40% of carriers.^1^ Core tumour types in LFS are osteosarcoma, soft tissue sarcoma, early-onset breast cancer, adrenocortical carcinomas, leukaemias and CNS (central nervous system) tumours.^4 5^

The management of cancers in LFS is challenging given their exquisite radiosensitivity^6^ and metachronous nature. Therefore, cancer prevention^7^ and early detection are the most effective means of improving cancer outcomes, with early detection being coupled with early curative treatment or interception strategies. Risk-reducing mastectomy is offered for women with LFS^8^,^10^ and cancer early detection through annual clinical review and imaging surveillance.^8^,^11^ In the UK, the current standard of care for adults with LFS, introduced in 2021, is annual: clinical review, MRI brain (MRB) and whole-body MRI (WB-MRI) with similar guidelines adopted worldwide,^8^,^11^ in response to studies showing detection of early cancers by WB-MRI.^12^,^14^ Such a programme has not been formally evaluated in a long-term longitudinal setting; therefore, we undertook evaluation of our single-centre annual WB-MRI and MRB surveillance in adult individuals with LFS.

## Methods

We performed a retrospective single-centre study of adults with LFS undergoing annual WB-MRI between 1 January 2012 and 23 January 2024, and MRB surveillance between 30 August 2017 and 23 January 2024 using clinical and imaging records. All individuals had a *TP53* (likely) PGV or proven somatic mosaicism. Censoring date for all clinical information was 1 May 2024.

WB-MRI surveillance scans comprised T1 coronal and STIR (short tau inversion recovery) sequences without contrast enhancement, with additional diffusion-weighted imaging (DWI) sequences being adopted into practice from 2017. The first brain surveillance scan was with gadolinium contrast enhancement, with subsequent scans being without. The MRB surveillance scans comprised axial T1 and T2, coronal FLAIR (fluid-attenuated inversion recovery) and DWI, postgadolinium axial and coronal T1, and sagittal T1 volume sequences of the head.

Statistical analyses were performed using Excel (V.16.93, Microsoft) and Prism (V.10.3, GraphPad Software).

## Results

75 individuals (49 female, 26 male) were included in this study. 72 had a constitutional *TP53* PGV and three somatic mosaicism. 38 individuals were cancer naïve at enrolment while 37 had a previous cancer diagnosis. The median age at first WB-MRI was 36.7 years (mean=37.9, range=18.9–66.2). 325 (cancer naïve, n=179; previous cancer, n=146) surveillance WB-MRI scans were performed. Individuals underwent a mean of 4.3 scans (95% CI 3.8 to 4.9, median=4.0, range=1–12.0). Mean interval between WB-MRIs was 15.4 months (95% CI 14.7 to 16.2, median=13.4, range=3.5–44.5), with three individuals each found to have had WB-MRI scans at intervals of less than 6 months. Of the 325 WB-MRI scans, 213 (65.5%) were reported as normal. 112 (out of the 325) WB-MRI scans demonstrated abnormalities; 23/112 scans showing lesions of concern and 89/112 scans showing benign incidental findings prompting additional clinical review.

Nine cancers (in nine patients) were identified on nine of the 23 scans (39.1%) which were concerning for cancer. Seven out of nine were primary cancers and two were metastatic cancers demonstrable on WB-MRI scan. Three prevalent cancers in three individuals were identified on their first surveillance (index) WB-MRI which had not been diagnosed at enrolment. Mean time from previous normal WB-MRI surveillance scan for the remaining six individuals was 14.9 months (95% CI 12.4 to 17.4, median=14.7, range=12.6–19.0) (table 1).

**Table 1:** Cancers identified during study including WB-MRI surveillance detected and interval cancers following normal preceding WB-MRI scan

Patient number (sex)	Previous cancer diagnosis and age (range in years)	WB-MRI detected versus interval cancer	Time from previous normal WB-MRI scan (months)	Final diagnosis, age (range in years)	Early (stage 1/2) or late (stage 3/4) cancer seen at diagnosis	Outcome following diagnosis (months/years) at censoring
1 (F)	Breast cancer (bilateral, metachronous), 21–30 years	WB-MRI detected	14.6	Lung adenocarcinoma, 41–50 years	Early	Alive, 1.8 years
2 (F)	N/A	WB-MRI detected	15.8	Follicular thyroid cancer, 31–40 years	Early	Alive, 5.0 years
3 (F)	Breast cancer (bilateral, metachronous), 21–30 years	WB-MRI detected	14.7	Leiomyosarcoma*, 41–50 years	Early	Alive, 2.1 years
4 (F)	Osteosarcoma, 11–20 years; breast cancer (bilateral, synchronous), 31–40 years	WB-MRI detected	12.6	Leiomyosarcoma, 31–40 years	Early	Alive, 4.5 years
5 (M)	N/A	WB-MRI detected	19.0	Metastatic leiomyosarcoma of unknown primary, 21–30 years	Late	Deceased, 1.4 years
6 (M)	Primary sarcomas, 11–20 and 31–40 years	WB-MRI detected	12.7	Metastatic iliac osteosarcoma, 41–50 years	Late	Deceased, 1.5 months
7 (M)	Prostate cancer, 61–70 years	WB-MRI detected	Index scan	Non-small cell lung carcinoma, 61–70 years	Early	Deceased, 2.7 years
8 (F)	Liver sarcoma, 11–20 years; breast cancer, 21–30 years	WB-MRI detected	Index scan	Recurrent liver sarcoma, 21–30 years	Early	Deceased, 1.8 years
9 (F)	Ovarian cancer, 31–40 years; sarcoma, 41–50 years; breast cancer, 41–50 years	WB-MRI detected	Index scan	Abdominal wall sarcoma, 51–60 years	Early	Alive, 8.6 years
10 (M)	N/A	Interval diagnosis	8.2	Acute lymphoblastic leukaemia (ALL), 21–30 years	Early	Deceased, 6.8 years
10 (M)	N/A	Interval diagnosis	11.2	High-grade sarcoma, 31–40 years	Late	Deceased, 3.2 years
11 (M)	Leiomyosarcoma, 41–50 years	Interval diagnosis	7.8	High-grade glioma, 41–50 years	Late	Deceased, 1.0 year
12 (F)	N/A	Interval diagnosis	5.5	Pancreatic adenocarcinoma, 51–60 years	Late	Deceased, 3.0 months
13 (M)	N/A	Interval diagnosis	4.9	Chronic myeloid leukaemia (CML), 61–70 years	Late	Deceased, 2.0 years
14 (F)	N/A	Interval diagnosis	10.1	Breast cancer†, 21–30 years	Early	Alive, 6.7 years
15 (M)	N/A	Interval diagnosis	7.4	Gastro-oesophageal adenocarcinoma, 51–60 years	Late	Deceased, 2.0 years
16 (F)	Breast cancer, 41–50 years	Interval diagnosis	11.5	Metastatic mediastinal sarcoma, 41–50 years	Late	Deceased, 2.5 years

* Imaging suggested enlarging uterine fibroids; however, final diagnosis was driven by clinical suspicion.

† Detected on dedicated breast MRI surveillance.

WB-MRI, whole-body MRI.

Eight interval cancers were diagnosed in seven individuals following a previously reported normal surveillance WB-MRI scan (seven clinical presentations, one surveillance breast MRI detected breast cancer; mean time from previous normal WB-MRI was 8.3 months (95% CI 6.3 to 10.3, range=4.9–11.5), table 1). We analysed the performance of WB-MRI surveillance as a cancer early detection tool across a range of patient/cancer statuses with early cancer detection (stage 1/2 disease) in cancer-naïve individuals showing specificity=95.5%, negative predictive value (NPV)=99.4%, sensitivity=50.0% and positive predictive value (PPV)=11.1% when excluding haematological and breast cancers (table 2).

**Table 2:** Summary of surveillance WB-MRI performance

	Sensitivity (%)	Specificity (%)	PPV (%)	NPV (%)
All cancer types				
Whole cohort, excluding prevalent diagnoses*	42.86	95.50	30.00	97.38
All stage cancers (excluding haematological and breast)				
Cancer naïve at enrolment	40.00	95.40	20.00	98.22
Whole cohort excluding prevalent diagnoses	54.55	95.50	30.00	98.34
Whole cohort including prevalent diagnoses	64.29	95.50	39.13	98.34
Early cancers (stage 1/2, excluding haematological and breast)				
Cancer naïve at enrolment	50.00	95.48	11.11	99.41
Whole cohort excluding prevalent diagnoses	80.00	95.58	22.22	99.67
Whole cohort including prevalent diagnoses	87.50	95.58	33.33	99.67

* Prevalent diagnosis—cancer that was detected on the first WB-MRI scan.

NPV, negative predictive value; PPV, positive predictive value; WB-MRI, whole-body MRI.

89 (out of 325) (27.4%) surveillance WB-MRIs in 72/75 individuals (96.0%) identified 65 different benign appearing lesions (11 individuals had more than one). This resulted in 53 additional investigations (US=24; MRI=18; CT=8; X-ray=3) in 46 individuals with no cancers being identified.

32 individuals (23 female, 9 male; constitutional *TP53* PGV, n=32; 15/32 cancer naïve) underwent 53 surveillance MRB scans. Median age at first MRB scan was 37.0 years (mean=37.1, range=20.3–53.6). Five (out of 53) scans (9.4%) prompted further investigation and neurosurgical multidisciplinary team review, with one dysembryoplastic neuroepithelial tumour detected in an asymptomatic patient with surveillance MRB. Two of these five scans also demonstrated concomitant benign incidental findings. A further seven scans, nine (out of 53) scans in total (17.0%), identified benign incidental findings requiring further follow-up imaging.

## Discussion

We report the findings from the largest single-centre study detailing outcomes of annual WB-MRI surveillance in 75 adults with LFS over a 12-year period. 16 individuals developed 17 cancers; nine were detected by WB-MRI and eight were interval cancers. All but two interval cancers were diagnosed at a late stage. 10 individuals had died from cancer (four detected, six interval) at the time of reporting, including two which were detected early (stages 1 and 2). Two individuals had late-stage cancers (both sarcomas) detected on WB-MRI surveillance scans, with one individual developing metastases from a previous osteosarcoma. After breast cancer, osteosarcomas and soft tissue sarcomas are the most common cancer types in LFS.^1 15^ Given the stark discrepancy in 5-year survival of early (>80%) versus late-stage (18%) disease for soft tissue sarcoma,^16^ it is critical for an early detection strategy to include their early detection. The absence of a current cancer prevention strategy and the inherent challenges of WB-MRI for sarcoma detection (eg, distal limbs) further compound this need. This study demonstrates the impact and severity of osteosarcoma and soft tissue sarcoma on individuals with LFS and highlights the need for improved and novel early detection methods in this cohort, as these lesions are often detected late and demonstrate poor survival rates despite treatment.

Seven individuals developed eight interval cancers not detected by WB-MRI surveillance; however, this comprised four cancers that WB-MRI would not be considered adept at identifying early: chronic myeloid leukaemia, acute lymphoblastic leukaemia, pancreatic adenocarcinoma and gastrointestinal malignancy. One breast cancer was not detected on WB-MRI and was considered an interval cancer for the purpose of WB-MRI evaluation; however, the cancer was detected on annual breast MRI surveillance as per guidance,^8^ further highlighting its importance in LFS surveillance alongside WB-MRI. Retrospective review of an individual’s WB-MRI scans following sarcoma diagnosis (patient 10) demonstrated the presence of subcutaneous oedema as the only notable preceding finding before sarcoma development which may inform future radiological reporting. We included diffusion-weighted sequences from 2017 to augment diagnostic accuracy and believe the evolution of protocols with experience is paramount to improving surveillance outcomes in LFS.

The interval high-grade glioma in our study predated current guidance recommending MRB surveillance^8 9 11^ and echoes the lack of sensitivity of WB-MRI in detecting brain tumours as noted by other authors.^15 17^ Our data for annual MRB surveillance are too immature for a full analysis given its later adoption compared with WB-MRI; individual patient choice in view of the anxiety additional surveillance imaging may induce; and whereby abnormalities detected on MRB surveillance prompt referral to the regional neurosurgical unit limiting access to imaging data. However, we note that 1/32 individuals had an MRB detected intracranial tumour.

In our study, the over-riding finding was that where a scan was considered negative for cancer, then this result mostly held true (specificity >95%, NPV >97%). Thus, WB-MRI is a useful tool for excluding cancer; however, it is much less effective in the discrimination of abnormal findings detected, demonstrated by low sensitivities/PPVs across subcohorts. While this would be unlikely to be acceptable in a population-based screening programme, given the high lifetime cancer risk in LFS, the threshold for clinical and radiological concern for reporting lesions is low as the main driving concern is not missing a cancer in this high-risk population. This is, however, at the expense of the additional follow-up imaging required and the associated anxiety for these individuals. Ongoing learning and multidisciplinary case discussion may help guide future practice regarding additional investigations following WB-MRI surveillance. Other studies have reported outcomes from single baseline WB-MRI scans in individuals with LFS^12 14 18^ with cancer detection in 7–16% of individuals. Considering longitudinal surveillance, Villani *et al*^14^ reported surveillance-detected tumours occurring in 19/59 individuals (including children) undergoing clinical, biochemical and imaging surveillance over a median of 32-months; here, almost half of individuals developed benign lesions only (eg, adenomas) with one developing breast ductal carcinoma in situ. Excluding these diagnoses to make them more comparable with WB-MRI surveillance alone, their cancer detection rate is similar to other baseline reports at 17%, and this report of 12% in a longitudinal series.

This study solely comprises an adult population where the occurrence of non-specific benign findings and other non-LFS-related epithelial neoplasms will be greater than in the paediatric population. With outcomes from paediatric cancers improving, and the discovery of *TP53* PGVs of lower penetrance for adult cancers such as R337H (*TP53* c.1010G>A p.Arg337His)^19^ and P152L (*TP53* c.455C>T p.Pro152Leu),^20^ this presents a challenge for effective early detection surveillance in LFS that has improved specificity without compromising sensitivity and is an area requiring further research.

Novel means of cancer early detection have the potential to supplement WB-MRI surveillance in the LFS cohort. We demonstrate that four out of nine individuals with cancers detected by WB-MRI had died at the time of reporting and all within 3 years of diagnosis. A recent study investigating genomic alterations in cfDNA in individuals with LFS as an initial means of early detection did demonstrate a significantly higher PPV (67.5%) as compared with our WB-MRI experience, without significant compromise of NPV (96.5%). Here, two individuals developed changes in the cfDNA pan-cancer methylation and genome-wide fragmentation signals at 7 and 20 months, respectively, prior to their clinical presentation of osteosarcoma.^21^

We report the findings from the longest and largest single-centre experience of annual WB-MRI for cancer early detection in adults with LFS, demonstrating a high and acceptable level of cancer exclusion but poor specificity prompting supplementary imaging. Annual WB-MRI and clinical review is the current standard of care guidance for cancer early detection in adults with LFS, but is challenging. Therefore, there is an urgent need to develop novel means of early detection that may either complement imaging as a surveillance tool, or enable imaging to form part of the diagnostic pathway where an abnormal early detection marker is seen in this high-risk population.

## Data Availability

This work uses data provided by patients and collected by the National Health Service (NHS) as part of their clinical care. The patients have not given written consent for their individualised data to be shared publicly beyond that which is included within the manuscript and, therefore, due to the sensitive nature of the research, supporting data are not available.
